# Antibacterial Properties of Medicinal Plants From Pakistan Against Multidrug-Resistant ESKAPE Pathogens

**DOI:** 10.3389/fphar.2018.00815

**Published:** 2018-08-02

**Authors:** Muhammad Faraz Khan, Huaqiao Tang, James T. Lyles, Rozenn Pineau, Zia-ur-Rahman Mashwani, Cassandra L. Quave

**Affiliations:** ^1^Department of Botany, Pir Mehr Ali Shah Arid Agriculture University, Rawalpindi, Pakistan; ^2^Center for the Study of Human Health, Emory University College of Arts and Sciences, Atlanta, GA, United States; ^3^Department of Botany, Faculty of Basic and Applied Sciences, University of the Poonch, Rawalakot, Pakistan; ^4^Department of Dermatology, Emory University School of Medicine, Atlanta, GA, United States; ^5^Antibiotic Resistance Center, Emory University, Atlanta, GA, United States

**Keywords:** medicinal plants, biofilm, quorum sensing, *Zanthoxylum armatum*, delta-toxin

## Abstract

Local people in the Sudhnoti district of Pakistan share a rich practice of traditional medicine for the treatment of a variety of ailments. We selected nine plants from the Sudhnoti ethnopharmacological tradition used for the treatment of infectious and inflammatory disease. Our aim was to evaluate the *in vitro* anti-infective potential of extracts from these species against multidrug-resistant (MDR) ESKAPE (*Enterococcus faecium, Staphylococcus aureus, Klebsiella pneumoniae, Acinetobacter baumanii, Pseudomonas aeruginosa*, and *Enterobacter* species) pathogens. Plant specimens were collected in the Sudhnoti district of Pakistan and vouchers deposited in Pakistan and the USA. Dried bulk specimens were ground into a fine powder and extracted by aqueous decoction and maceration in ethanol. Extracts were assessed for growth inhibitory activity against ESKAPE pathogens and biofilm and quorum sensing activity was assessed in *Staphylococcus aureus*. Cytotoxicity to human cells was assessed via a lactate dehydrogenase assay of treated human keratinocytes (HaCaTs). Four ethanolic extracts (*Zanthoxylum armatum, Adiantum capillus-venaris, Artemisia absinthium*, and *Martynia annua*) inhibited the growth of MDR strains of ESKAPE pathogens (IC_50_: 256 μg mL^−1^). All extracts, with the exception of *Pyrus pashia* and *M. annua*, exhibited significant quorum quenching in a reporter strain for *S. aureus agr* I. The ethanolic extract of *Z. armatum* fruits (Extract 1290) inhibited quorum sensing (IC_50_ 32–256 μg mL^−1^) in *S. aureus* reporter strains for *agr* I-III. The quorum quenching activity of extract 1290 was validated by detection of δ-toxin in the bacterial supernatant, with concentrations of 64–256 μg mL^−1^ sufficient to yield a significant drop in δ-toxin production. None of the extracts inhibited *S. aureus* biofilm formation at sub-inhibitory concentrations for growth. All extracts were well tolerated by human keratinocytes (LD_50_ ≥ 256 μg mL^−1^). Chemical analysis of extract 1290 by liquid chromatography-Fourier transform mass spectrometry (LC-FTMS) revealed the presence of 29 compounds, including eight with putative structural matches. In conclusion, five out of the nine selected anti-infective medicinal plants exhibited growth inhibitory activity against at least one MDR ESKAPE pathogen at concentrations not harmful to human keratinocytes. Furthermore, *Z. armatum* was identified as a source of quorum quenching natural products and further bioassay-guided fractionation of this species is merited.

## Introduction

Antimicrobial resistance (AMR) represents one of the most concerning threats to global health, and new anti-infectives are needed to overcome it (Thabit et al., 2015). AMR occurs when microorganisms are able to survive in the presence of drugs that would normally inhibit their growth (Founou et al., 2017). It is estimated that approximately 700,000 people currently die each year from AMR infections, and this number is projected to reach 10 million annually by the year 2050 (O'Neill, 2016). The Infectious Disease Society of America has highlighted a small group of bacteria (*Enterococcus faecium*, *Staphylococcus aureus*, *Klebsiella pneumoniae*, *Acinetobacter baumanni*, *Pseudomonas aeruginosa, and* *Enterobacter* species), hereafter referred to as ESKAPE pathogens (Boucher et al., 2009), which exhibit resistance to many antibiotic drug classes and are a high priority for drug discovery. Reinvigorating the pipeline of anti-infectives in development is more critical today than ever, as increasingly hard-to-treat bacteria continue to emerge (Pewtrusts.org., 2018). Further, novel anti-infectives that act through new mechanisms of action are needed (Schroeder et al., 2017). Plants represent a promising source of natural products in efforts to identify bioactive compounds (Cowan, 1999; Rossiter et al., 2017).

### Role of plant natural products in anti-infective drug discovery

Plants used in traditional medicinal practices against infections have been found to inhibit growth and virulence of various microbes (Ahmad and Beg, 2001; Kumar et al., 2006; Bibi et al., 2011; Cioch et al., 2017). Traditional medicine is one of the most easily available treatment methods in developing countries, with approximately 80% of the population in some regions using traditional medicine to meet their primary healthcare needs (WHO, 2002; Maroyi, 2013). Plants synthesize a diverse array of chemicals, known as secondary metabolites, as an adaptation for self-defense and communication with other organisms in their ecosystems (Harborne and Baxter, 1995). These secondary metabolites possess many advantages for anti-infective drug development, including being generally bioactive, being drug-like and metabolite-like, and harboring potential for synergy with other secondary metabolites as part of a plant's multicomponent defense system (Harvey et al., 2015).

Most studies on the anti-infective activity of plant extracts have focused on their growth inhibitory potential, and without great success; there are no classic (bacteriostatic or bactericidal) antibiotics derived from plant secondary metabolites on the market. Investigation into alternative anti-infective mechanisms of action can open new avenues in drug development to combat antibiotic resistance (Schroeder et al., 2017), and natural products may serve as a critical reservoir of antibiotic adjuvants to overcome resistance mechanisms (Wright, 2017). One target of great interest, for example, is biofilm production. Biofilms are surface-associated microbial communities that can survive high concentrations of treatment by physically occluding drug entry via the biofilm matrix and through reduction in the rate of cellular metabolism (Wu et al., 2015). Inhibiting the formation of biofilms could allow biofilm-associated infections to be more effectively resolved with antibiotic treatment. For example, treatment with an ellagic acid glycoside-rich blackberry extract improved the ability of several functionally distinct classes of antibiotics to significantly reduce the number of biofilm-associated *S. aureus* cells on a medical device (Quave et al., 2012).

Another target of increasing interest is bacterial virulence (Otto, 2004, 2014). In *S. aureus*, for example, virulence is mediated by quorum sensing, a two-component cell signaling system of stimuli and response based on population density. Many components of *S. aureus* quorum sensing controlled virulence factor production pathways are encoded by the accessory gene regulator (*agr*) gene locus, of which four subtypes exist: *agr* I-IV. Quorum quenching (QQ) by *agr* inhibition has been shown to result in an inhibition of *S. aureus* virulence in a number of studies, including those showing inhibition with plant secondary metabolites (Quave et al., 2015; Muhs et al., 2017).

### Plants are critical to anti-infective traditional medicine practices in pakistan

The majority of people in Pakistan rely on medicinal plants for the treatment of ailments; 12% of Pakistani flora are used in medicine and more than 300 medicinal plants are traded for this purpose (Shinwari, 2010). Due to its geographic position, ethnopharmacological practices in Pakistan stem from Ayurvedic, Islamic and local traditional medicine (Shaikh and Hatcher, 2005). Traditional Unani medicine is also popular among a large segment of the country (Qureshi et al., 2009). The northern part of Pakistan, including the State of Jammu and Kashmir, is rich with medicinal plants, providing the main supply of raw herbal products to herbal manufacturers (*davakhanas*) and the international market (Shinwari, 2010). Nine medicinal plant species were selected for this study based on observations of traditional use for the treatment of infectious and inflammatory diseases during fieldwork (Fluck and Jaspersen Schib, 1941) and literature searches on their reported traditional uses and known activities. We selected species with no prior or limited antibacterial analysis (e.g., only examined for growth inhibition via agar well or disc diffusion methods). All selected species are present in abundant populations in the collection region and none are listed as threatened or endangered. The aims of our study were to evaluate the impact of extracts from the following medicinal plants on (1) the growth of ESKAPE pathogens; (2) the biofilm and quorum sensing capacity of *S. aureus;* (3) mammalian cytotoxicity in human keratinocytes; and to (4) chemically characterize the most bioactive extract(s). We focused our biofilm and quorum quenching assays on *S. aureus* as a proof of concept to demonstrate other potential pathways through which anti-infective plant extracts may act; we did not have the appropriate biofilm deficient mutant control strains or reporter model systems for the other ESKAPE pathogens available to test in this study. We have summarized the ethnopharmacological basis for the selection of each species below:

- ***Adiantum capillus-veneris*** L., Pteridaceae, is a fern commonly found in moist temperate regions of the Himalayas. It is commonly used as an aqueous infusion to treat urinary tract infections (UTIs). It is also used as an astringent, demulcent, antitussive and diuretic (Ishaq et al., 2014).
- ***Artemisia absinthium*** L., Asteraceae, is used topically as a paste of aerial plant parts for pruritus and inflammatory and infectious skin disorders (Khan and Khatoon, 2008). A number of *Artemisia* species have traditional uses, and several have been utilized for the development of drugs against malaria and typhoid (Hayat et al., 2009).
- ***Berberis lycium*** Royle, Berberidaceae, is a prickly shrub traditionally used to treat diarrhea, cholera and piles (Malik et al., 2017). Other indications include jaundice, diabetes, eye infections, fractured bones, internal wounds and diarrhea (Ali et al., 2015). Bark infusions are traditionally used for oral infections, toothaches and earaches (Abbasi et al., 2010).
- ***Gentiana olivieri*** Griseb., Gentianaceae is an herb found abundantly in the alpine and sub-alpine areas of the Himalayas. Its traditional medical use is as a root decoction for the treatment of urinary rentention (Ali and Qaiser, 2009; Bano et al., 2014).
- ***Martynia annua*** L., Martyniaceae, is an herbaceous, erect, glandular herb commonly known as “bichoo.” It grows in locations with ample organic matter, i.e., landfills. Leaf paste is traditionally topically applied to infected wounds and skin conditions. Leaf juice is used for wound healing and in gargles for sore throat (Santram and Singhai, 2011; Dhingra et al., 2013).
- ***Nerium oleander*** L., Apocynaceae, is commonly known as “kneer.” It is used for the treatment of oral and topical infections (Hussain and Gorsi, 2004). Young branches are used as chewing sticks to treat oral infections by indigenous communities in Himalayan valleys.
- ***Pyrus pashia*** Buch.-Ham. ex D.Don, Rosaceae, is a woody plant in the rose family with edible fruit. It is locally used as a laxative and to treat gastrointestinal, cardiovascular and respiratory ailments (Abbasi et al., 2013; Janbaz et al., 2015).
- ***Swertia chirata*** Buch.-Ham. ex C.B. Clarke, Gentianaceae, is widely used as a whole plant infusion by local people for the treatment of hepatitis, inflammation and digestive diseases. Other indications include chronic fever, malaria, skin disease and bronchial infections (Kumar and Van Staden, 2016).
- ***Zanthoxylum armatum*** DC., Rutaceae, locally known as “timber” has been used by local people as a chewing stick to treat dental infections and oral sores. The fruits and bark of the plant are also reportedly used to treat cancer and digestive ailments such as cholera and dysentery (Ahmad et al., 2014; Alam and us Saqib, 2017).

## Materials and methods

### Plant collection and identification

**Figure 1:**
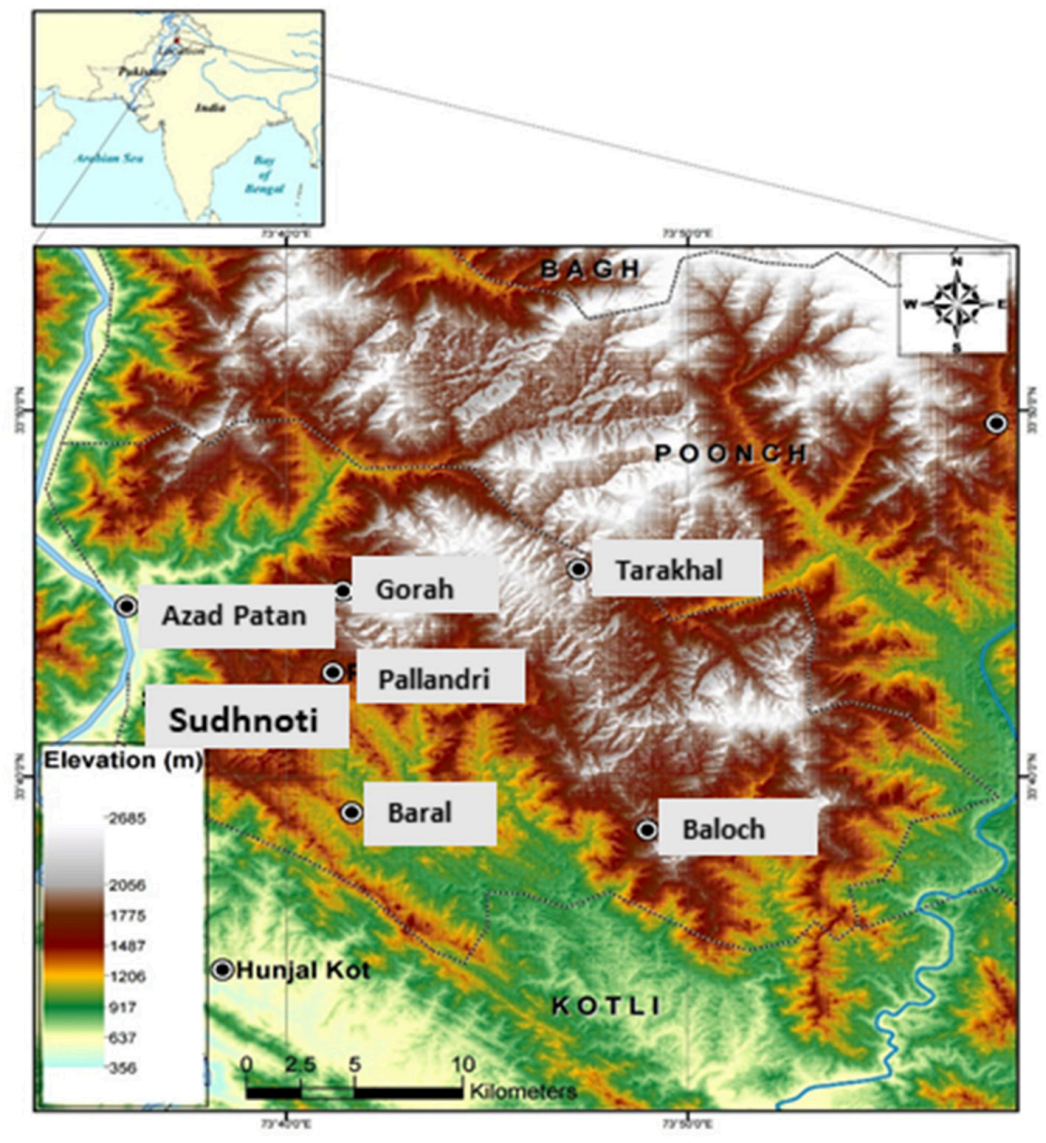
Map of the study region: Sudhnoti district in Northern Pakistan.

Plant material was collected from the Sudhnoti district in Northern Pakistan (Figure 1) during an ethnobotanical survey conducted from Fall 2015 to Spring 2017. Nine medicinal species were selected for collection based on their potential against infectious diseases as determined by traditional use and depth of available research reported in the literature. Plant collections were made on private land with permission of local owners and community representatives following the World Health Organization Guidelines on good agricultural and collection practices for medicinal plants (WHO, 2003). Bulk plant material was separated into parts, which were then clipped into small pieces and dried in the shade. Voucher specimens of each species were prepared for identification and deposited in herbaria in Pakistan and the USA (PMAS-UAAR and GEO).

Identification of plant specimens was carried out with the assistance of Dr. Rehmatullah Qureshi (PMAS-UAAR, Pakistan) and Dr. Tharangamala Samarakoon (GEO: Emory University Herbarium, USA). Dry plant material was imported to the USA under an import permit issued by the United States Department of Agriculture (Permit Number: PCIP-17-00110). Plant material was stored at −80°C upon arrival. Voucher specimens (listed in Table 1) deposited at GEO were digitized for open access viewing on a web-based platform (SERNEC, 2017)^1^. The Plant List was consulted for family assignment and confirmation of latest botanical names (TPL, 2017), and the Angiosperm Phylogeny Group IV was used for family assignments (APG, 2016).

**Table 1:** Plant species tested with a description of parts used, extraction solvent, and percent yield obtained for each extract.

**Family**	**Species**	**Voucher ID**	GEO accession number^*^	**Part used**	**Extract ID**	**Extract solvent**	**Percent yield (%)**
Apocynaceae	*Nerium oleander* L.	FK-102	22,199	Leaves	1292 1298	EtOH dH_2_O	14.72 15.81
Asteraceae	*Artemisia absinthium* L.	FK-106	22,195	Aerial parts	1308 1311	EtOH dH_2_O	17.68 11.89
Berberidaceae	*Berberis lycium* Royle	FK-105	22,196	Root	1289 1295	EtOH dH_2_O	13.94 7.5
Gentianaceae	*Gentiana olivieri* Griseb.	FK-104	22,197	Aerial parts	1293 1299	EtOH dH_2_O	9.68 6.54
	*Swertia chirata* Buch.-Ham. ex C.B. Clarke	FK-103	22,198	Whole plant	1291 1297	EtOH dH_2_O	6.67 20.25
Martyniaceae	*Martynia annua* L.	FK-735	–	Fruit	1310 1312	EtOH dH_2_O	7.92 2.41
Pteridaceae	*Adiantum capillus-veneris* L.	FK-108	22,194	Whole plant	1294	EtOH	3.19
Rosaceae	*Pyrus pashia* Buch.-Ham. ex D. Don	FK-101	22,200	Fruit	1309 1322	dH_2_O EtOH	18.44 2.38
Rutaceae	*Zanthoxylum armatum* DC.	FK-73	22,201	Fruit	1290 1296	EtOH dH_2_O	7.50 7.27

* *GEO: Emory University Herbarium*.

### Preparation of extracts

Following shipment and storage at −80°C, plant materials were dried at 35°C in a dehumidifying drier for 3 days and ground into fine powder (2 mm mesh size) with a Thomas Scientific Wiley Mill (Swedesboro, NJ). Retention vouchers of chopped and ground material were prepared for future reference. For the preparation of organic crude extracts, plant powder was macerated for 72 h in 1 L flasks at a 1:10 ratio (w/v) in 95% ethanol with daily agitation. Macerates were filtered, the marc collected, and residual plant material subjected to a repeat extraction in 95% ethanol. Solvent was removed by rotary evaporation at ≤40°C. Extracts were redissolved in deionized water (dH_2_O), shell frozen in a dry ice-acetone bath, and then lyophilized overnight on a Labconco FreeZone 2.5 Lyophilizer (Kansas City, MO). Dry extracts were stored in scintillation vials at −20°C.

Aqueous extracts were prepared by boiling 30–40 g of plant material (1:10 ratio w/v) for 20 min in dH_2_O on a hot plate, followed by centrifugation and vacuum filtration. Aqueous extracts were then evaporated on a rotary evaporator and subsequently lyophilized as described above. Organic and aqueous crude extracts of all the plant samples were dissolved in DMSO or dH_2_O, respectively, to a stock concentration of 10 mg mL^−1^ prior to testing in biological assays.

### Antibacterial testing

#### Bacterial strains and cultures

Twenty-two strains from seven bacterial species were used in this study (Table 2). These strains comprise drug resistant bacteria recognized by the American Society of Microbiology as ESKAPE pathogens: *Enterococcus faecium* (EU-49, EU-44), *Staphylococcus aureus* (UAMS-1, UAMS-929, LAC, AH1677, AH430, AH1747, AH1872, NRS225, NRS232, NRS242, NRS249, NRS385), *Klebsiella pneumoniae* (EU-32, CDC-76), *Acinetobacter baumannii* (CDC-33, EU-24), *Pseudomonas aeruginosa* (CDC-54, PAO1), *Enterobacter cloacae* (CDC-32), and *Enterobacter aerogenes* (CDC-7). Antibiotic resistance profiles and other strain characteristics are reported in Table 2. All strains were streaked from freezer stock onto tryptic soy agar (TSA) plates and incubated at 37 °C overnight before making overnight liquid cultures in cation-adjusted Mueller-Hinton broth (CAMHB) or tryptic soy broth (TSB). All antibacterial tests were conducted in triplicate and repeated at least once on a separate day, and included appropriate positive (antibiotics, biofilm inhibitors, or virulence inhibitors) and negative controls, including vehicle and media controls.

**Table 2:** ESKAPE pathogens tested and their corresponding antibiotic resistance profiles as reported by the source provider (BEI Resources or CDC AR Bank) or as determined by antibiotic disc diffusion test (for AMC, IPM, PIP, RA, SXT, and TET) following CLSI breakpoints.

**Species**	**Strain ID**	**Alternate ID**	Antibiotic resistance profile^*^	**Other characteristics**
*Acinetobacter baumannii*	CDC-33	AR-BANK #0033	CAZ, CIP, CRO, CTX, DOR, FEP, GEN, IPM, LVX, MEM, SAM, SXT, TOB, TZP	reduced susceptibility, elevated carbapenem MICs
	EU-24	Naval-81; NR-17786	PIP, SXT, TET, TZP	
*Enterobacter aerogenes*	CDC-7	AR-BANK #0007	AMC, AMP, ATM, CAZ, CFZ, CIP, CRO, CTX, ETP, FEP, FOX, LVX, SAM, TET	reduced susceptibility, elevated carbapenem MICs
*Enterobacter cloacae*	CDC-32	AR-BANK #0032	AMC, AMP, ATM, CAZ, CFZ, CRO, CTX, ETP, FEP, FOX, IPM, MEM, SAM, SXT, TZP	reduced susceptibility, elevated carbapenem MICs
*Enterococcus faecium*	EU-44	HM-959; Strain 513	AMC, RA, SXT, TET, TZP	
	EU-49	NR-31915; Strain E0164	AMC, GEN, TET, SXT, TZP, VAN	
*Klebsiella pneumoniae*	EU-32	NR-15410	AMC, PIP, SXT, TZP	Contains β-lactamase *K. pneumoniae* carbapenemase (blaKPC) gene
	CDC-76	AR-BANK #0076	AMC, AMP, CAZ, CFZ, CTX, DOR, FEP, FOX, GEN, IPM, MEM, SAM, SXT, TOB, TZP	reduced susceptibility, elevated carbapenem MICs
*Pseudomonas aeruginosa*	CDC-54	AR-BANK #0054	CAZ, FEP, GEN, IPM, LVX, MEM, TOB, TZP	
	PAO1	AH-0071		
*Staphylococcus aureus*	LAC	AH0845	OXA, PIP	PFGE: USA300; CA-MRSA
	UAMS-1			Osteomyelitis clinical isolate; prototype biofilm isolate
	UAMS-929			Isogenic Δ*sarA* mutant of UAMS-1, biofilm deficient control strain
	AH1677			*agr* I yfp reporter strain (chloramphenicol resistant)
	AH430			*agr* II yfp reporter strain
	AH1747			*agr* III yfp reporter strain
	AH1872			*agr* IV yfp reporter strain
	NRS232	HT20020065	ERY^I^, GEN, PEN	*agr* I*, egc+, (lukS-lukF PVL)+, hlg+;* associated with necrotising pneumonia
	NRS242	HT20020238	ERY^I^, PEN	*agr* IV*, egc+, (lukS-lukF PVL)+, (lukE-lukD)+, hlgv*+; associated with impetigo
	NRS245	HT20020320; NR-46038	CIP, OXA, PEN	*agr* I*, sea+, sed+, (lukE-lukD)+, hlgv+;* associated with impetigo; SCCmec type IV
	NRS249	HT20020341; NR-46042	CIP, CLI, ERY, GEN, OXA, PEN	*agr* I*, sea+, (lukE-lukD)+, hlgv+ associated with native valve endocarditis;* SCCmec type IV
	NRS385	95938; NR-46071	CIP, CLI, ERY, GEN, LVX, SXT, TET	*agr* I; PFT is USA500, MLST is ST8, spa type is YHGCMBQBLO, SCCmec IV; *sea*+, *seb*+; HA-MRSA

* *Resistance: AMC, amoxicillin-clavulanic acid; AMP, ampicillin; ATM, aztreonam, CAZ, ceftazidime; CFZ, cefazolin; CIP, ciprofloxacin; CLI, clindamycin; CRO, ceftriaxone; CTX, cefotaxime; DOR, doripenem; ETP, ertapenem; FEP, cefepime; FOX, cefoxitin; GEN, gentamicin; IPM, imipenem; LVX, levofloxacin; MEM, meropenem; OXA, oxacillin, PEN, penicillin; PIP, piperacillin; RA, rifampicin; SAM, ampicillin-sulbactam; SXT, trimethoprim-sulfamethoxazole; TET, tetracycline; TOB, tobramycin; TZP, piperacillin-tazobactam; VAN, vancomycin. Any antibiotics denoted with an ^I^ indicates intermediate resistance*.

#### Growth inhibition assay

Extracts were examined for growth inhibitory activity following the guidelines of the Clinical and Laboratory Standards Institute for broth microdilution testing (CLSI, 2013). Overnight cultures were diluted in Cation-adjusted Muller Hinton broth (CAMHB) based on their optical density (OD_600nm_) to a confluence of 5 × 10^5^ CFU mL^−1^, confirmed by plate counts. Assays were performed in 96-well plates (Greiner Bio-One International, CELLSTAR 655-185). Plates were incubated for 18–24 h, depending upon the test recommendations for the species (CLSI, 2013), at which point they were read at an OD_600nm_ in a Cytation-3 multimode plate reader (Biotek). The percent of growth inhibition was calculated using a previously described formula that takes into account extract color (Quave et al., 2008). The IC_50_ was defined as the concentration required to achieve ≥50% inhibition of growth, as determined by OD. The MIC was defined as the concentration required to achieve an optically clear well (equivalent to the IC_90_, or concentration required to achieve ≥90% inhibition of growth as determined by OD_600nm_).

#### Biofilm inhibition assay

The biofilm inhibiting activity of extracts was assessed using biofilm media with human plasma in a static microtiter plate crystal violet assay as previously described (Quave et al., 2012) against *S. aureus* UAMS-1 with its isogenic biofilm deficient mutant UAMS-929 as a positive control (Beenken et al., 2003). A previously reported biofilm inhibiting extract, 220D-F2 from *Rubus ulmifolius* (Quave et al., 2012), was used as a positive chemical control.

#### Agr reporter assay for quorum sensing inhibition

Quorum sensing inhibition activity also known as quorum quenching of the plant extracts was assessed against all four accessory gene regulator (*agr*) subtypes of *S. aureus*. Fluorescent reporter strains (AH1672, AH430, AH1747, and AH1872, Table 2) were used as previously described (Quave and Horswill, 2014; Muhs et al., 2017). Controls included vehicles (DMSO and dH_2_O) and 224C-F2, a previously reported quorum sensing inhibitor extract (Quave et al., 2015). Quorum quenching activity was expressed as a percent vehicle value of the reporter strains' yellow fluorescent protein (YFP) signal. The IC_50_ and IC_90_ represent the concentration necessary to inhibit ≥50 or ≥90% of signaling, respectively, in comparison to the vehicle control. All extracts were initially screened against an *agr* I reporter strain at 256 μg mL^−1^. Dose response curves were obtained by 2-fold serial dilutions of treatments at a concentration range of 8–256 μg mL^−1^.

#### δ-toxin inhibition

Levels of δ-toxin (also known as δ-hemolysin) production in extract-treated culture supernatant were quantified by HPLC using a previously described protocol (Quave and Horswill, 2018) in order to determine if the *agr* inhibitory activity of extract 1290 (EtOH extract of Z. *armatum*) observed in the reporter assay corresponded with a decline in exotoxin production. The amount of δ -toxin present in the supernatant of treated and untreated culture samples was quantified using high toxin producing strains of *S. aureus*: NRS 225, NRS 232, NRS 242, NRS 249, NRS385 and LAC (Table 2). Extract 224C-F2 was used as a positive control (Quave et al., 2015).

### Mammalian cytotoxicity assay

Human skin keratinocytes (HaCaTs) and a lactate dehydrogenase (LDH) test kit (G-Biosciences, St. Louis, MO) were used to assess the potential cytotoxicity of extracts as previously described (Quave et al., 2015). All extracts were tested at a concentration of 100 μg mL^−1^. Extract 1290 was further evaluated at a concentration range of 8–1,024 μg mL^−1^. Percent DMSO v/v for all tests was <2%.

### Chemical characterization

#### High performance liquid chromatography (HPLC)

EtOH extracts of *Z. armatum, A. capillus-veneris* and *A. absinthium* were characterized by HPLC. The HPLC method was adapted from a previously published method (Bhatt et al., 2017). An Agilent Eclipse XDB-C18 4.6 × 250 mm, 5 μm analytical column with compatible guard column at 25°C was used for analysis. Extracts were prepared at 10 mg mL^−1^ in 70:20:10 ACN:MeOH:dH_2_O. A 20 μL sample injection was eluted at a flow rate of 1 mL min^−1^ using a mobile phase consisting of (A) 0.1% formic acid in dH_2_O, (B) 0.1% formic acid in ACN. The linear gradient had initial conditions of 98:2 A:B at 0 min, changing to 90:10 A:B at 19 min, then to 85:15 A:B at 59 min, and reaching 0:100 A:B at 100 min; this was maintained until 125 min, then the column was returned to initial conditions 98:2 A:B and held for 9 min. Data were collected with a diode array detector from 190 to 600 nm for all samples.

#### Mass spectrometry

LC-FTMS was performed on the bioactive ethanol extract of *Z. armatum* (1290) using a Thermo Scientific LTQ-FT Ultra MS equipped with a Shimadzu SIL-ACHT and Dionex 3600SD HPLC pump. For chromatography, the same sample preparations, HPLC column, and mobile phases were used as in the previously described HPLC method. Data were acquired in MS^1^ mode scanning from an m/z of 150–1,500 in negative and positive ESI (electrospray ionization) mode and processed with Thermo Scientific Xcalibur 2.2 SP1.48 (San Jose, CA). The capillary temperature was 275.0°C, sheath gas of 60, source voltage 5.00 kV, source current 100.0 μA, and the capillary voltage −19.0 or +32.0 V for negative and positive modes, respectively. Putative formulas were determined by performing isotope abundance analysis on the high-resolution mass spectral data with X-caliber software and reporting the most closely matching empirical formula. Database searches were performed using Scifinder (American Chemical Society) and the Dictionary of Natural Products (Taylor & Francis Group). The databases were reviewed for compounds identified from the genus *Zanthoxylum* with molecular masses corresponding to the LC-FTMS data.

### Statistical analyses

All data were analyzed using a two-tailed Student's *t*-test as calculated by GraphPad Prism 7 software (GraphPad Software, La Jolla, CA). DMSO or dH_2_O treated (vehicle control) cultures were used as a vehicle control and were compared to those treated with extract for all statistical analyses. *P* < 0.05 were considered statistically significant. All assays and other experiments were performed in triplicate or quadruplicate, and repeated on two separate days.

## Results

### Antibacterial activity

#### Extracts exhibit limited growth inhibitory activity in ESKAPE pathogens

**Figure 2:**
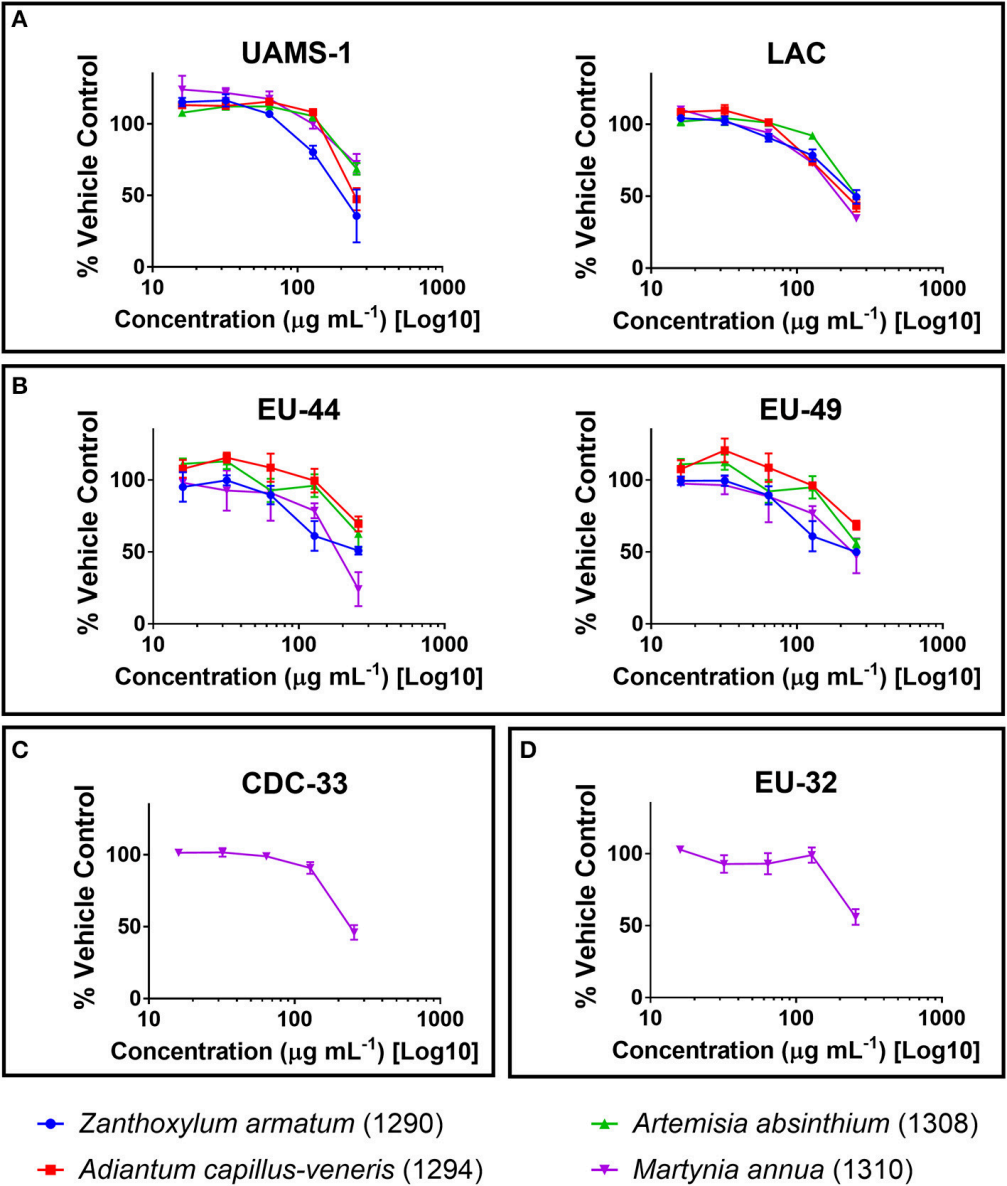
Growth inhibitory activity of ethanolic extracts that reached an IC_50_ against **(A)** *Staphylococcus aureus*; **(B)** *Enterococcus faecium*; **(C)** *Acinetobacter baumannii*; and **(D)** *Klebsiella pneumoniae*, reported as percent of the vehicle-treated control (DMSO).

Crude extracts of the selected plant species (Table 1) were screened against ESKAPE pathogens at a concentration of 256 μg mL^−1^ in broth microdilution assays. None of the organic extracts exhibited an IC_50_ against *Enterobacter aerogenes* (CDC-7), *E. cloacae* (CDC-32), or *Pseudomonas aeruginosa* (CDC-54 or PAO1) (Table 3), and the aqueous extracts did not exhibit an IC_50_ against any strain. The ethanolic extract of *M. annua* was the only extract to exhibit an IC_50_ against an *Acinetobacter baumannii* strain (CDC-33). This *M. annua* extract also exhibited an IC_50_ for one strain of *E. faecium* (EU-44), one strain of *Klebsiella pneumoniae* (EU-32), and both strains of *Staphylococcus aureus* (LAC and UAMS-1) tested for growth inhibition. *Z. armatum* and *A. capillus-veneris* ethanolic extracts both exhibited an IC_50_ against both strains of *E. faecium* and *S. aureus* tested. The other plant species tested (*N. oleander, B. lycium, G. oliverieri* and *P. pashia*) did not meet the threshold of 50% inhibition of growth at the screening concentration (256 ug mL^−1^). Dose dependent inhibition of *S. aureus* (LAC and UAMS-1) and *Enterococcus faecium* (EU44 and EU49) growth was demonstrated by the ethanol extracts of *Z. armatum, A. capillus-venaris, A. absinthium*, and *M. annua*, with IC_50_ values of 256 μg mL^−1^ for *S. aureus* and *E. faecium*, respectively. The only extract to exhibit dose dependent inhibition of *A. baumannii* (CDC-33) and *K. pneumoniae* (EU-32) was *M. annua*, with an IC_50_ of 256 μg mL^−1^ (Figure 2). None of the extracts exhibited an MIC (>90% inhibition) at the maximum concentration tested (256 μg mL^−1^).

**Table 3:** Summary of organic (EtOH) and aqueous extracts exhibiting growth inhibition ≥50% (IC_50_) against ESKAPE pathogens when screened at 256 μg mL^−1^.

**Plant Species**	**Extract ID**	***A. baumannii***		***E. aerogenes***	***E. cloacae***	***E. faecium***		***K. pneumoniae***		***P. aeruginosa***		***S. aureus***	
		**CDC-33**	**EU-24**	**CDC-7**	**CDC-32**	**EU-44**	**EU-49**	**EU-32**	**CDC-76**	**CDC-54**	**PAO1**	**LAC**	**UAMS-1**
*Nerium oleander*	1292	**–**	**–**	**–**	**–**	**–**	**–**	**–**	**–**	**–**	**–**	**–**	**–**
	1298	**–**	**–**	**–**	**–**	**–**	**–**	**–**	**–**	**–**	**–**	**–**	**–**
*Artemisia absinthium*	1308	**–**	**–**	**–**	**–**	+	+	**–**	**–**	**–**	**–**	**–**	+
	1311	**–**	**–**	**–**	**–**	**–**	**–**	**–**	**–**	**–**	**–**	**–**	**–**
*Berberis lycium*	1289	**–**	**–**	**–**	**–**	**–**	**–**	**–**	**–**	**–**	**–**	**–**	**–**
	1295	**–**	**–**	**–**	**–**	**–**	**–**	**–**	**–**	**–**	**–**	**–**	**–**
*Gentiana olivieri*	1293	**–**	**–**	**–**	**–**	**–**	**–**	**–**	**–**	**–**	**–**	**–**	**–**
	1299	**–**	**–**	**–**	**–**	**–**	**–**	**–**	**–**	**–**	**–**	**–**	**–**
*Swertia chirata*	1291	**–**	**–**	**–**	**–**	**–**	**–**	**–**	**–**	**–**	**–**	+	**–**
	1297	**–**	**–**	**–**	**–**	**–**	**–**	**–**	**–**	**–**	**–**	**–**	**–**
*Martynia annua*	1310	+	**–**	**–**	**–**	+	**–**	+	**–**	**–**	**–**	+	+
	1312	**–**	**–**	**–**	**–**	**–**	**–**	**–**	**–**	**–**	**–**	**–**	**–**
*Adiantum capillus–veneris*	1294	**–**	**–**	**–**	**–**	+	+	**–**	**–**	**–**	**–**	+	+
*Pyrus pashia*	1309	**–**	**–**	**–**	**–**	**–**	**–**	**–**	**–**	**–**	**–**	**–**	**–**
	1322	**–**	**–**	**–**	**–**	**–**	**–**	**–**	**–**	**–**	**–**	**–**	**–**
*Zanthoxylum armatum*	1290	**–**	**–**	**–**	**–**	+	+	**–**	**–**	**–**	**–**	+	+
	1296	**–**	**–**	**–**	**–**	**–**	**–**	**–**	**–**	**–**	**–**	**–**	**–**

“+”: *growth inhibition ≥50% in comparison to vehicle control; “−“: <50% growth inhibition in comparison to vehicle control.*

*Dose-response curves for active extracts are reported in Figure 2*.

#### Extracts exhibit limited to no biofilm inhibitory activity in *S. aureus*

**Figure 3:**
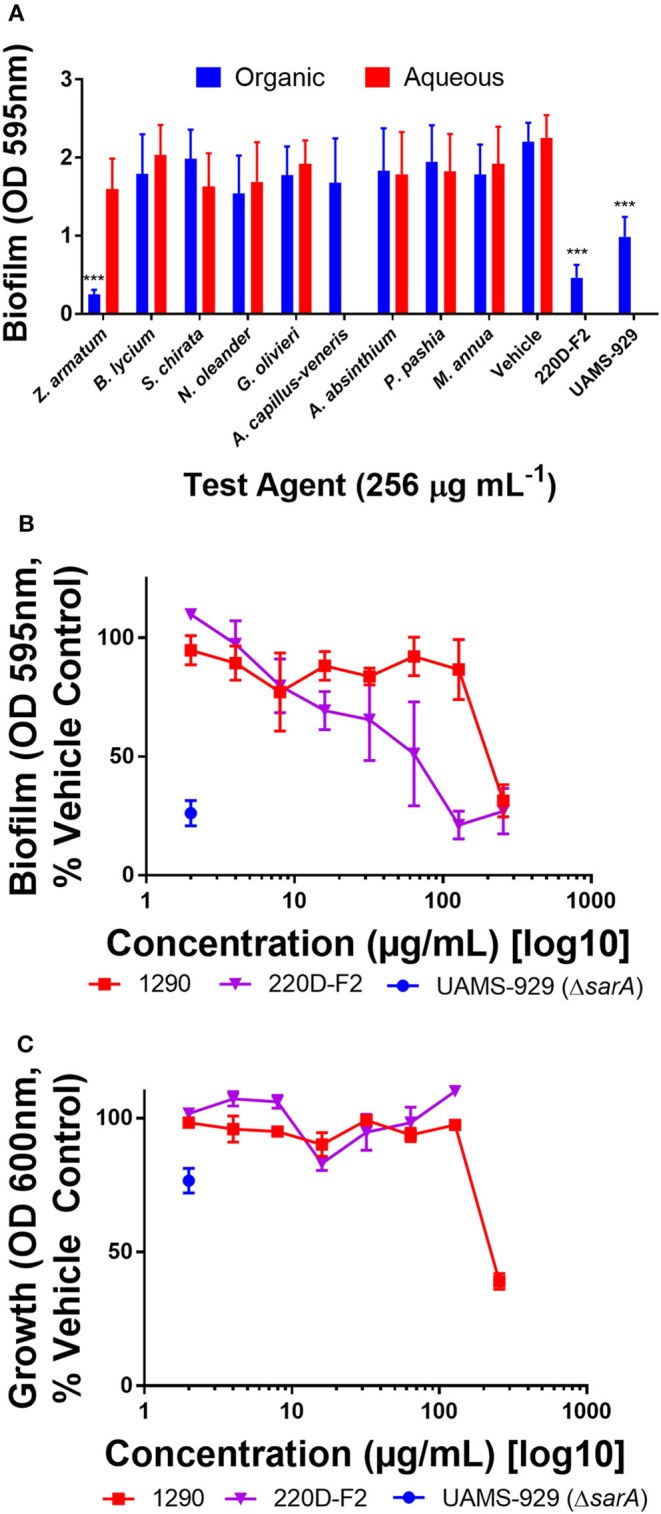
Impact of extracts on biofilm formation in *S. aureus* (UAMS-1). **(A)** Biofilm inhibition of extracts at 256 μg mL^−1^. (**B)** Biofilm dose-response study with extract 1290, *Z. armatum* fruit ethanolic extract. **(C)** Growth dose response study with extract 1290, *Z. armatum* fruit ethanolic extract. Positive controls included the biofilm deficient isogenic sarA mutant of UAMS-1 (UAMS-929) and a previously documented biofilm inhibiting extract, 220D-F2 (Quave et al., 2012). Significance was determined in comparison to the vehicle control, with ^*^p < 0.05, ^**^p < 0.01, and ^***^p < 0.001.

In our static microtiter plate crystal violet assay, modest biofilm inhibitory activity was noted among organic extracts when screened at 256 μg mL^−1^. The extracts had no major (>50% inhibition) impact on biofilm formation, with the exception of extract 1290 (*Z. armatum*) (Figure 3A). However, further analysis revealed that the observed anti-biofilm activity (Figure 3B) was due to overall growth inhibition at this dose (Figure 3C), rather than biofilm targeted activity.

#### Extracts exhibit modest to high quorum quenching activity in *S. aureus*

**Figure 4:**
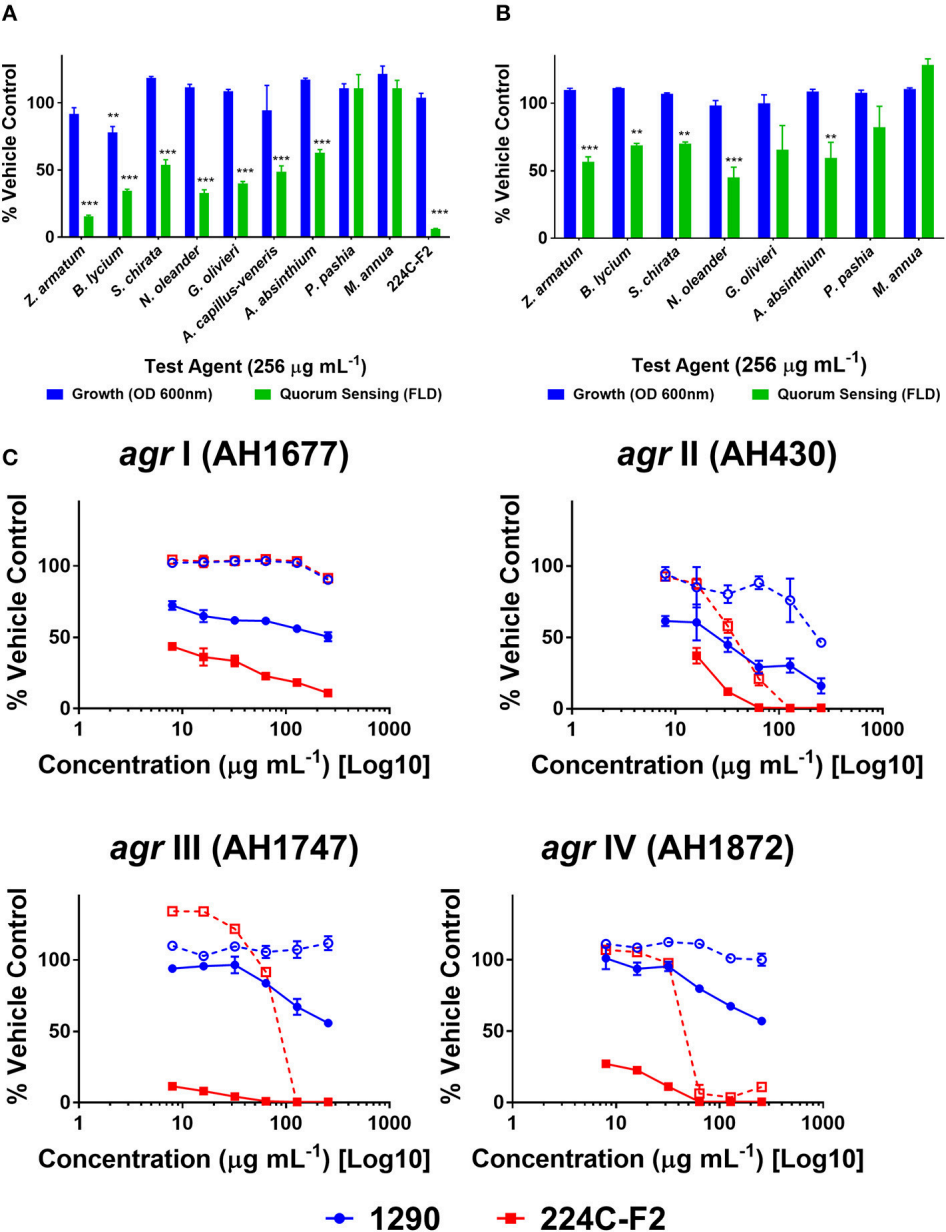
Quorum quenching activity of extracts on *S. aureus agr* reporter strains. Extracts were screened against an *agr* I reporter strain of *S. aureus* (AH1677) at a concentration of 256 μg mL^−1^. Growth was measured by OD, and quorum sensing activity by FLD. Vehicle (water and DMSO) and positive extract (224C-F2) were included: **(A)** ethanolic extracts **(B)** aqueous extracts. Significance was determined in comparison to the vehicle control, with ^*^p < 0.05, ^**^p < 0.01, and ^***^p < 0.001. **(C)** The active extract 1290 (*Z. armatum* EtOH fruit extract) was examined in dose response assays against *agr* I-IV reporters in comparison to the positive control (224C-F2), reported as %FLD of vehicle control for solid lines and %OD for growth in dashed lines.

Quorum quenching (QQ) activity was investigated using four reporter strains of *S. aureus agr* subtypes (*agr* I-IV). QQ activity was revealed by a screen of the extracts at 256 μg mL^−1^ in an *agr* I reporter strain. Modest, but statistically significant, inhibition of quorum sensing was observed in tests on *B. lycium, S. chirata, N. oleander, G. olivieri, A. capillus-veneris*, and *A. absinthium* ethanol extracts (Figure 4A). Similarly, modest and significant inhibition of quorum sensing was observed in some of the aqueous extracts, including for *Z. armatum, B, lyceum, S. chirata, N. oleander* and *A. absinthium* (Figure 4B). Neither the ethanolic or aqueous extracts of *P. pashia* or *M. annua* exhibited any quorum quenching activity in the screen against *agr* I. The ethanolic extract of *Z. armatum* (1290), the most active extract, was subsequently tested against all four *agr* subtypes in a set of dose response experiments (Figure 4C). The extract inhibited quorum sensing in all four *agr* types at sub-MIC concentrations, and yielded an IC_50_ of 256, 32, and 256 μg mL^−1^ in *agr* types I, II and III, respectively. No IC_50_ was reached for *agr* IV at the maximum concentration tested.

#### Extract 1290 blocks production of virulence factor δ-toxin in *S. aureus*

**Figure 5:**
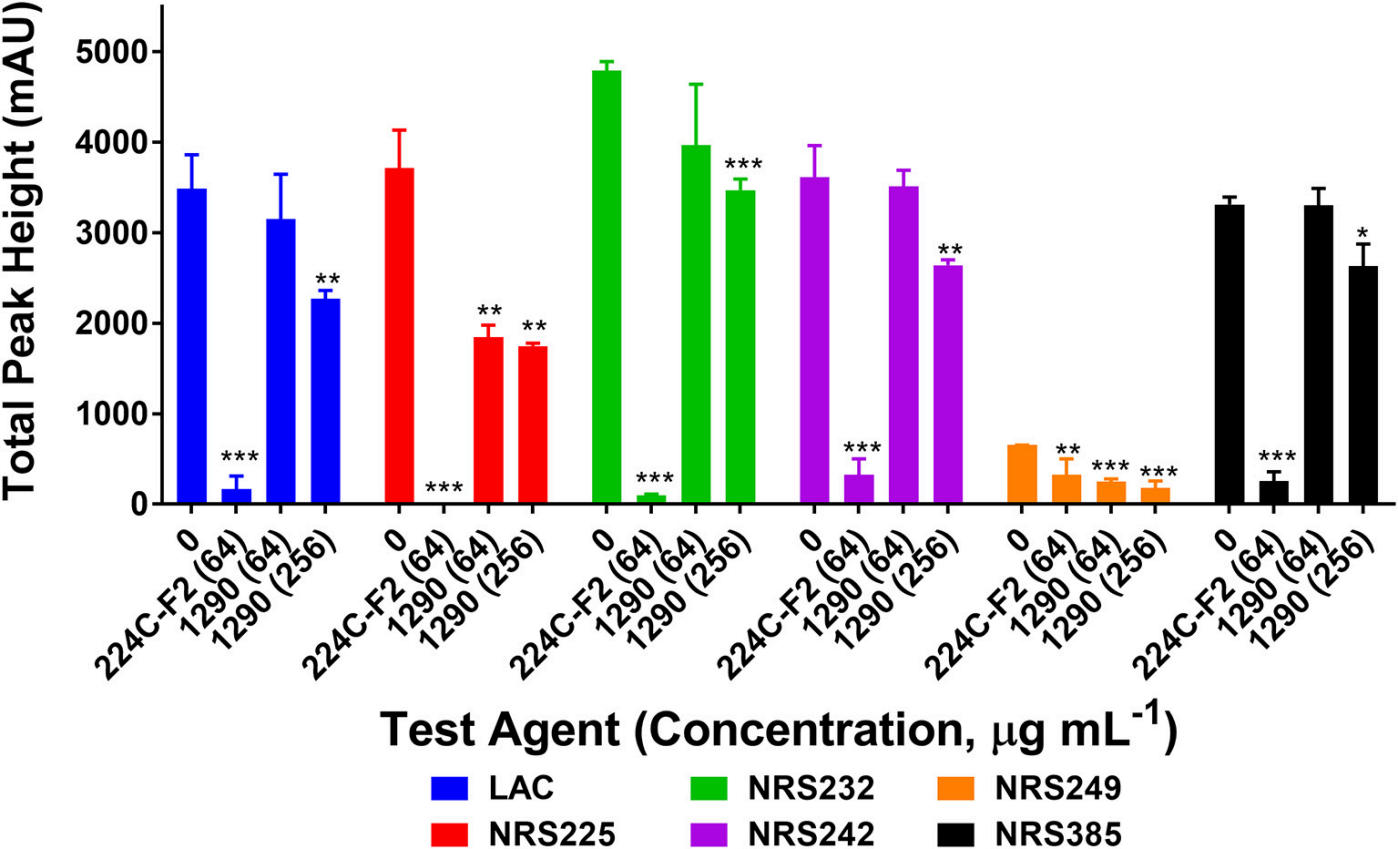
Impact of the ethanolic extract of *Zanthoxylum armatum* fruits on δ-toxin production (measured by HPLC) in six strains of hypervirulent *Staphylococcus aureus* (Table 2). Significance was determined in comparison to the vehicle control (DMSO), with ^*^p < 0.05, ^**^p < 0.01, and ^***^p < 0.001. 224C-F2, a bioactive fraction of *Castanea sativa* leaf extract (Quave et al., 2015), was used as a positive control (tested at 64 μg mL^−1^).

To confirm the quorum sensing inhibitory activity of extract 1290 observed in the reporter model (Figure 4), we assessed its ability to inhibit δ-toxin production at concentrations of 64 and 256 μg mL^−1^. To this end, six strains of *S. aureus* known to be high-level producers of δ-toxin were grown with extract 1290 or vehicle, and the cell supernatant harvested for analysis by HPLC. The extract significantly inhibited toxin production at doses of 64 and 256 μg mL^−1^ against two and six of the six strains studied, respectively (Figure 5).

### Extracts are nontoxic to human keratinocytes

**Figure 6:**
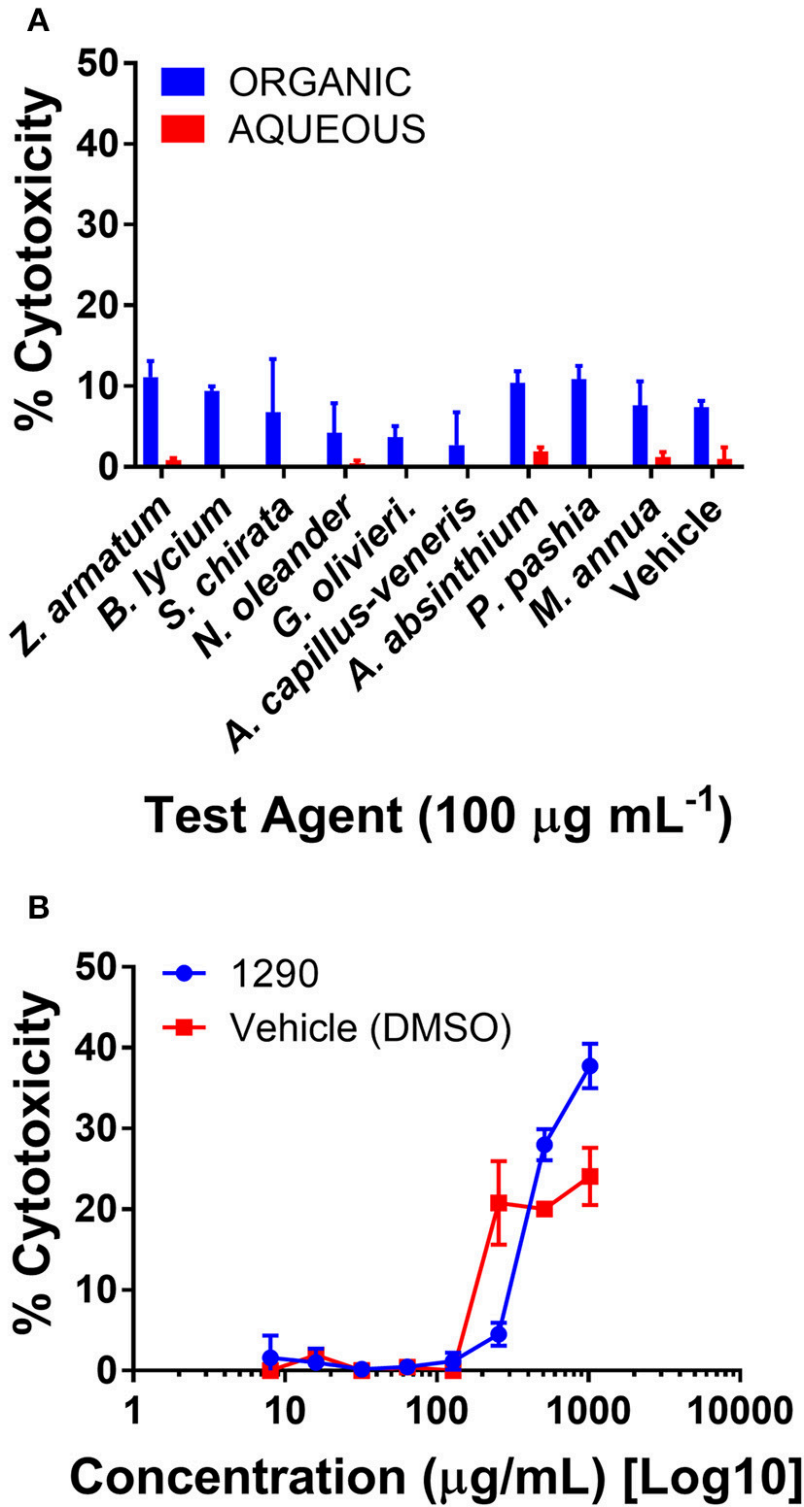
Cytotoxicity of extracts in a human keratinocyte (HaCaT) cell line by LDH assay for cell viability. **(A)** Organic and aqueous extracts were screened at 100 μg mL^−1^. Significance was determined in comparison to the vehicle control (DMSO), with ^*^p < 0.05, ^**^p < 0.01, and ^***^p < 0.001. **(B**) Extract 1290 was examined by dose response study from 8 to 1,024 μg mL^−1^.

Human skin keratinocytes (HaCaTs) were exposed to the aqueous and organic extracts to assess potential cytotoxic effects on mammalian cells. All extracts were well tolerated by the cell line when tested at a screening concentration of 100 μg mL^−1^ (Figure 6A). Due to its activity in antibacterial assays, extract 1290 was further examined in a dose-response study at a range of 8–1, 024 μg mL^−1^, and was found to exhibit slight toxicity over the vehicle control at the highest doses of 512 and 1,024 μg mL^−1^, but no IC_50_ was reached at these concentrations (Figure 6B).

### Chemical characterization of active extracts

**Figure 7:**
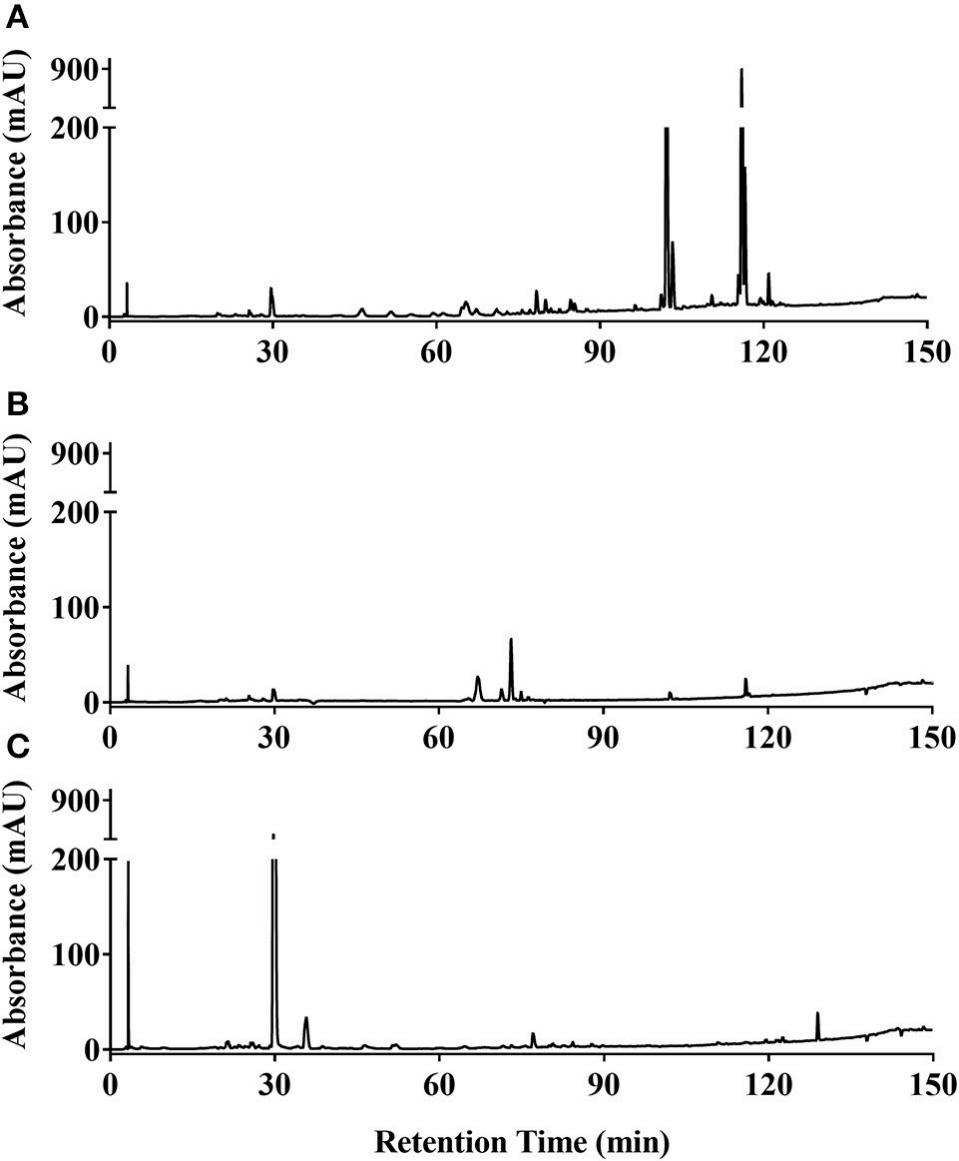
HPLC chromatograms at 254 nm of ethanolic extracts of **(A)** *Zanthoxylum armatum* fruits (1290); **(B)** *Adiantum capillus-veneris* whole plant (1,294); and **(C)** *Artemisia absinthium* aerial parts (1,308).

**Figure 8:**
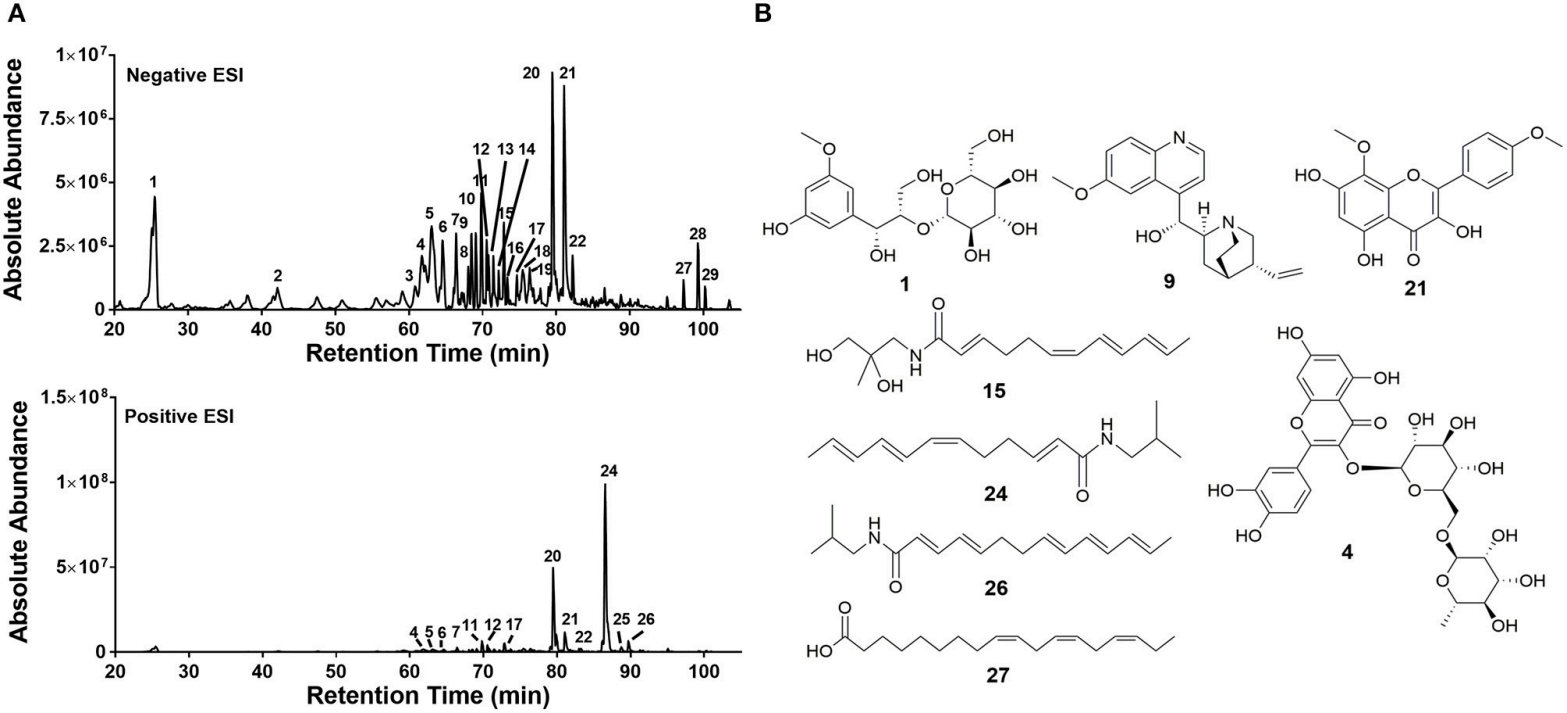
Chemical characterization of extract 1290. **(A)** LC-FTMS ESI negative and positive base peak chromatograms for 430D-F5. All peaks correspond to data presented in Table 4. **(B)** Putative structural matches are listed by peak number. Peak **1** was determined to be C_16_H_24_O_10_ and the putative structural match is threo-3-methoxy-5-hydroxy-phenylpropanetriol-8-O-β-D-glucopyranoside. Peak **4** was determined to be C_27_H_30_O_16_ and the putative structural match is 3-[[6-O-(6-deoxy-α-L-mannopyranosyl)-β-D-glucopyranosyl]oxy]-2-(3,4-dihydroxyphenyl)-5,7-dihydroxy-4H-1-benzopyran-4-one. Peak **9** was determined to be C_20_H_24_N_2_O_2_ and the putative structural match is 6'-methoxy-(8α,9R)-cinchonan-9-ol. Peak **15** was determined to be C_16_H_25_N O_3_ and the putative structural match is N-(2,3-dihydroxy-2-methylpropyl)-2,6,8,10-dodecatetraenamide. Peak **21** was determined to be C_17_H_14_O_7_ and the putative structural match is 3,5,7-trihydroxy-8-methoxy-2-(4-methoxyphenyl)-4H-1-benzopyran-4-one. Peak **24** was determined to be C_16_H_25_NO and putative structural match is N-(2-methylpropyl)-2,6,8,10-dodecatetraenamide. Peak **26** was determined to be C_18_H_27_NO and the putative structural match is N-(2-methylpropyl)-2,4,8,10,12-tetradecapentaenamide. Peak **27** was determined to be C_18_H_30_O_2_ and the putative structural match is 9,12,15-Octadecatrienoic acid.

Ethanol extracts of *Zanthoxylum armatum* (1290), *Adiantum capillus-veneris* (1294), and *Artemisia absinthium* (1308) were assessed by HPLC (Figure 7). As the most promising bioactive extract, *Z. armatum* was selected for further analysis by LC-FTMS, which revealed the presence of 29 major compounds with relative abundance >1%. Putative matches based on isotopic analysis and database searches were possible for 8 of these: (**1**) threo-3-methoxy-5-hydroxy-phenylpropanetriol-8-O-β-D-glucopyranoside, (**4)** 3-[[6-O-(6-deoxy-α-L-mannopyranosyl)-β-D-glucopyranosyl]oxy]-2-(3,4-dihydroxyphenyl)-5,7-dihydroxy-4H-1-benzopyran-4-one, (**9**) 6′-methoxy-(8α,9R)-cinchonan-9-ol, (**15**) N-(2,3-dihydroxy-2-methylpropyl)-2,6,8,10-dodecatetraenamide, (**21**) 3,5,7-trihydroxy-8-methoxy-2-(4-methoxyphenyl)-4H-1-benzopyran-4-one, (**24**) N-(2-methylpropyl)-2,6,8,10-dodecatetraenamide, (**26**) N-(2-methylpropyl)-2,4,8,10,12-tetradecapentaenamide, (**27**) 9,12,15-octadecatrienoic acid (Figure 8, Table 4).

**Table 4:** Negative and positive ESI Mass spectrometry (*m/z*) analysis of extract 1290; peaks with >1% relative abundance are listed.

**Peak #**	**RT (min)**	**(%) Relative abundance**		**Formula (Δ -ppm)^a^**	**Putative compounds**	**ESI Mode**	***m/z*^b^**	**MS^2^**
		**Neg.Mode**	**Pos.Mode**					
1	25.45	–	3.19	C_16_H_24_O_10_ (0.3)	threo-3-methoxy-5-hydroxy-phenylpropanetriol-8-O-β-D-glucopyranoside	+	**375.12897**, 766.27824	194.87242, 176.97254
2	42.14	2.48	0.49	C_14_H_28_O_9_ (2.9)	no matches	–	**340.17687**, 408.16466	294.03311, 276.07231
3	60.84	1.4	0.46	C_21_H_19_O_10_ (0.3)	no matches	–	431.09892, **499.08670**	310.97984, 340.98113
4	61.75	5.79	1.59	C_27_H_30_O_16_ (1.4)	3-[[6-O-(6-deoxy-α-L-mannopyranosyl)-β-D-glucopyranosyl]oxy]-2-(3,4-dihydroxyphenyl)-5,7-dihydroxy-4H-1-benzopyran-4-one	–	**609.14745**, 499.08701	300.91790, 299.92698
5	63.07	7.54	1.46	C_31_H_31_O_20_ (0.9)	no matches	–	**463.08914**, 723.14223	300.91790, 299.92698
6	64.55	3.7	0.99	C_21_H_19_ O_12_ (1.0)	no matches	–	**577.08306**, 927.18881	463.03392, 424.98160
7	66.39	3.29	1.34	C_21_H_19_O_11_ (0.8)	no matches	–	**447.09409**, 593.1531	283.92544, 327.00141
8	68.05	1.17	0.29	C_24_H_21_O_14_ (1.0)	no matches	–	**533.09481**, 601.08393	489.04098
9	68.49	2.26	0.47	C_20_H_24_N_2_O_2_ (−2.0)	6′-methoxy-(8α,9R)-cinchonan-9-ol	–	**326.19793**, 607.39844	280.04288, 236.09022
10	69.07	2.12	–	C_38_H_55_O_6_ (-1.9)	no matches	–	**607.39844**, 394.18629	298.97899, 561.12814
11	69.79	3.75	1.84	C_20_H_24_N_2_O_2_ (-1.9)	no matches	–	**324.1823**, 603.36732	278.00556, 220.01256
12	70.53	3.03	1.48	C_17_H_28_O_10_ (4.0)	no matches	–	**392.17037**, 603.36731	278.00556, 324.21714
13	71.46	1.64	0.49	C_17_H_30_O_10_ (1.4)	no matches	–	**394.18629**, 593.18978	376.00632, 349.99389
14	72.18	0.92	0.23	C_31_H_37_O_11_ (1.1)	no matches	–	**585.2353**, 635.35729	539.16004
15	72.83	–	1.87	C_16_H_25_NO_3_ (−0.5)	N-(2,3-dihydroxy-2-methylpropyl)-2,6,8,10-dodecatetraenamide	+	**278.17511**, 584.27451	204.89416, 162.92277
16	73.35	0.67	–	C_31_H_37_O_11_ (1.1)	no matches	–	**585.23543**, 327.21828	539.22769, 347.09425
17	74.67	0.91	0.29	C_18_H_33_O_5_ (0.3)	no matches	–	**329.23375**, 621.3777	229.0025, 211.03004
18	75.44	2.26	–	C_38_H_53_O_7_ (-2.7)	no matches	–	**621.37695**, 689.36582	575.01788, 296.03969
19	76.39	1.9	1.25	C_38_H_53_O_7_ (-2.9)	no matches	–	621.37677, 689.36591	575.01788, 296.03969
20	79.46	9.96	14.01	C_14_H_28_O_7_ (3.2)	no matches	–	**308.18728**, 605.38301	262.06560, 290.08820
21	81.05	–	4.65	C_17_H_14_O_7_ (-0.7)	3,5,7-trihydroxy-8-methoxy-2-(4-methoxyphenyl)-4H-1-benzopyran-4-one	+	**331.08107**, 280.19090	316.04065
22	82.21	1.18	–	C_25_H_29_O_8_ (0.6)	no matches	–	**457.18752**, 411.18216	411.05709, 389.12406
23	83.23	–	0.91	C_21_H_21_O_5_ (-0.1)	no matches	+	**353.13831**, 741.29147	322.00452, 190.94986
24	86.53	–	41.32	C_16_H_25_NO (-0.1)	N-(2-methylpropyl)-2,6,8,10-dodecatetraenamide	+	495.39285, **248.20077**	149.09491, 174.90641
25	88.74	–	1.12	C_30_H_57_O_8_ (2.7)	no matches	+	**545.40814**, 361.32182	527.25299, 284.11325
26	89.7	–	2.23	C_18_H_27_NO (0.05)	N-(2-methylpropyl)-2,4,8,10,12-tetradecapentaenamide	+	**274.21659**, 373.32187	174.98173, 132.92981
27	97.29	0.7	–	C_18_H_30_O_2_ (0.2)	9,12,15-Octadecatrienoic acid	–	555.44309, **277.21756**	233.1687, 259.12869
28	99.23	1.79	0.29	C_17_H_31_O_4_ (0.5)	no matches	–	**507.44336**, 299.22334	445.27496, 283.91006
29	100.2	0.52	0.28	C_36_H_63_O_4_ (1.2)	no matches	–	**559.47447**, 497.34979	321.05199, 255.13572

The corresponding chromatograms for negative and positive ESI are reported in Figure 8A, with putative structural matches reported in Figure 8B.

a *The empirical formula corresponds to the [M+H]^+^ or [M-H]^−^ ion as determined by the ionization mode*.

b *The reported ions correspond to the [M+H]^+^ or [M-H]^−^ ion as determined by the ionization mode. When multiple ions were formed, the number in bold font indicates the [M+H]^+^ or [M-H]^−^ and underwent MS^2^ analysis*.

## Discussion

The aim of this study was to examine extracts of plants selected based on traditional medicinal use for growth inhibitory, biofilm inhibitory, cytotoxicity, and quorum quenching activity in a series of *in vitro* assays. We selected a panel of clinically-relevant, multidrug-resistant human pathogens for these studies. All of the selected medicinal species exhibited some degree of anti-infective activity in our models, with the exception of *Pyrus pashia*. It is important to note that this does not rule out the potential efficacy of *P. pashia* in traditional medicine, where it is used as a laxative and for gastrointestinal disorders (Janbaz et al., 2015), as we did not specifically target laboratory models for these functions beyond inclusion of select gut pathogens, and are limited by exclusion of host-directed studies.

With regards to growth inhibition, it is not unexpected to see poor to no activity among extracts against the Gram-negative species tested. The presence of the outer membrane, which is difficult to penetrate, as well as constitutively overexpressed efflux pumps, make these pathogens much more difficult to target with antibiotics or herbal extracts than Gram-positive pathogens (Domalaon et al., 2018). Of the extracts evaluated, four exhibited activity against a Gram-positive pathogen, while the ethanolic extract of *Martynia annua* fruits (Extract 1310) was the only one to exhibit some activity against both Gram-positive and Gram-negative pathogens (Table 3). While the activity was modest (IC_50_ of 256 μg mL^−1^) against *Acinetobacter baumannii, Enterococcus faecium, Klebsiella pneumoniae* and *Staphylococcus aureus*, it is still worth further examination as the active consituent(s) may represent a small portion of the chemically complex crude extract. We recommend further studies focused on the bioassay-guided fractionation of this extract to determine if a refined fraction or isolated compound(s) can achieve an MIC at a therapeutically relevant concentration. The current literature on *M. annua* is focused on its efficacy in wound healing. To that end, studies demonstrate the efficacy of a 5% w/w ointment of *M. annua* methanolic fractions in dead space and burn wound models (Lodhi et al., 2016), a 0.5% w/w ointment flavonoid enriched fraction on healing in an *in vivo* diabetic wound model (Lodhi and Singhai, 2013), and fractions of ethanolic leaf extracts in rat excision and incision models (Santram and Singhai, 2011). To the best of our knowledge, this is the first report of antibacterial activity of this *M. annua* against ESKAPE pathogens.

Other species exhibiting dose-dependent growth inhibitory activity against *S. aureus* and *E. faecium* included *Zanthoxylum armatum, Artemisia absinthium*, and *Adiantum capillus-veneris* (Figure 2). A prior study on the antibacterial activity of *Z. armatum* extracts determined by disc diffusion and broth microtiter dilution methods reported MICs at very high doses, ranging from 10,000 to 200,000 μg mL^−1^, for a panel of 11 bacterial pathogens (Wazir et al., 2014). Other prior studies on the antibacterial activity of *Z. armatum* were restricted to disc diffusion assays and this methodology presents limitations in the comparison of one extract to another, and the establishment of IC_50_ or MIC values. However, disc diffusion results can be informative as a basic starting point to identifying species with potential antibacterial activity. In one study, the activity of an ethanolic extract of fruits was reported against a strain of *S. aureus* and *Salmonella typhi* (Hussain et al., 2017). In another study, the methanolic fraction of the fruits exhibited some activity when loaded at a dose of 500 μg disc^−1^ against *S. aureus, Streptococcus mutans, P. aeruginosa, Salmonella typhi, Enterococcus faecalis, Bacillus subtilis, Staphylococcus epidermidis*, and *Streptococcus pyogenes* (Nooreen et al., 2017). The antifungal activity of the fruit extract has also been reported, with the most activity demonstrated against *Trichophyton longifusis* at 200 μg mL^−1^ by agar dilution method (Alam and us Saqib, 2017).

The antibacterial activity of *Adiantum capillus-veneris* has been previously reported based on disc diffusion assays, with some activity noted against *Escherichia coli, Staphylococcus aureus*, and *Klebsiella pneumoniae*, but no MICs or mammalian cytotoxicity counter screen data is available (Ishaq et al., 2014). Likewise, the antibacterial activity of *Artemisia absinthium* has been broadly reported against a number of pathogens, including *Pseudomonas aeruginosa* and *Staphylococcus aureus*, see for example (Kordali et al., 2005; Valdés et al., 2008; Fiamegos et al., 2011; Moslemi et al., 2012).

Although none of the extracts inhibited biofilm formation in *S. aureus* at concentrations sub-inhibitory for growth (Figure 3), we did note significant *S. aureus* quorum quenching activity in all of the extracts tested, with the exception of the ethanolic extracts of *P. pashia* and *M. annua* (Figure 4A) and the aqueous extracts of *G. olivieri, P. pashia*, and *M. annua* (Figure 4B) in a *S. aureus agr* I reporter strain. Of all the extracts, *Z. armatum* exhibited the greatest potential for quorum quenching across all four *agr* types (Figure 4C), and this is the first report of its quorum quenching potential. In *S. aureus*, quorum sensing via a two component signaling system is the chief mediator of virulence, which includes the production of toxins that exert lytic activity on a number of human cell types (Grumann et al., 2014; Salam and Quave, 2018). Of these, δ-toxin production has been indicated as a very accurate measure of quorum sensing activity and bacterial virulence in general (Quave and Horswill, 2018). The δ-toxin gene, *hld*, is coded by RNAIII, the expression of which is directly regulated by quorum sensing (Benito et al., 2000). In addition to noting activity in *agr* fluorescent reporters, we also demonstrated the reduction in overall δ-toxin production by HPLC analysis following treatment of six *S. aureus* strains with extract 1290 of *Z. armatum* (Figure 5).

Our studies on *Z. armatum* fruit extract (1290) exhibited no substantial cytotoxicity (no IC_50_) at our maximum concentration tested (1,024 μg mL^−1^, Figure 6B), suggesting that use of this species is safe to human cells. Although the virulence assays were performed only on *S. aureus* strains in this study, further research on virulence mechanisms in other ESKAPE pathogens is merited. Furthermore, bioassay-guided fractionation of the extract—which exhibited significant, but modest, quorum quenching activity in *S. aureus*—could lead to improved activity with further refinement of the extract or isolation of active constituent(s).

## Conclusion

Our work has demonstrated the anti-infective potential of extracts from some selected Pakistani medicinal plants. Of the nine species tested, five exhibited some level of antibacterial activity in our growth inhibition model, none in staphylococcal biofilm inhibition, and seven in the *S. aureus* quorum quenching model. We suggest that future studies on the anti-infective potential of these medicinal plants also address the anti-biofilm and quorum quenching activity of the other ESKAPE pathogens, pending the development of appropriate control strains and reporter model systems.

Of greatest note for growth inhibitory studies, the fruit of *Martynia annua*, which is used in traditional medicine for skin infections and sore throat, exhibited the most broad-spectrum growth inhibitory activity against ESKAPE pathogens, including MDR strains of *Acinetobacter baumannii, Enterococcus faecium, Klebsiella pneumoniae*, and *Staphylococcus aureus* in an ethanol extract (1310). However, this growth inhibitory activity was modest, with an IC_50_ of 256 μg mL^−1^, and no MIC was determined at the maximum concentration tested. On the other hand, the fruit of *Zanthoxylum armatum*, which is used in traditional medicine to treat infectious diseases of the gut and oral cavity, demonstrated the most promising activity in terms of reducing staphylococcal virulence (IC_50_ 32–256 μg mL^−1^), as well as growth inhibition of the enteric pathogen *Enterococcus faecium* in an ethanol extract (1290). Chemical characterization of this extract revealed the presence of 29 major compounds, with putative matches for eight of these. The activity profile combined with absence of cytotoxicity (IC_50_ >1,024 μg mL^−1^) suggests both the presence of promising compounds for the potential development of anti-virulence approaches as well as validation of this traditional therapy for management of certain infectious diseases.

## Author contributions

MK collected the plant specimens, prepared extracts and performed the antibacterial and cell culture experiments. RP performed the biofilm experiments. MK, JL, and HT performed the chemical analysis of active extracts. HT performed δ-toxin analyses. CQ and ZM designed and directed the study. MK and CQ analyzed the data and wrote the manuscript. All authors read, revised and approved the final manuscript.

### Conflict of interest statement

The authors declare that the research was conducted in the absence of any commercial or financial relationships that could be construed as a potential conflict of interest.

## Footnotes

^1^Available online at: http://sernecportal.org/portal/s (Accessed Dec 1, 2017).
